# COVID-19 and Air Pollution: A Spatial Analysis of Particulate Matter Concentration and Pandemic-Associated Mortality in the US

**DOI:** 10.3390/ijerph19010592

**Published:** 2022-01-05

**Authors:** Brian H. Bossak, Samantha Andritsch

**Affiliations:** Department of Health and Human Performance, College of Charleston, Charleston, SC 29424, USA; andritschsc@g.cofc.edu

**Keywords:** COVID-19, pandemic, SARS-CoV-2, air pollution, spatial analysis, mortality, PM

## Abstract

In 2019, a novel coronavirus, SARS-CoV-2, was first reported in Wuhan, China. The virus causes the disease commonly known as COVID-19, and, since its emergence, it has infected over 252 million individuals globally and taken the lives of over 5 million in the same time span. Primary research on SARS-CoV-2 and COVID-19 focused on understanding the biomolecular composition of the virus. This research has led to the development of multiple vaccines with great efficacy and antiviral treatments for the disease. The development of biomedical interventions has been crucial to combating this pandemic; additionally, environmental confounding variables that could have exacerbated the pandemic need further assessment. In this research study, we conducted a spatial analysis of particulate matter (PM) concentration and its association with COVID-19 mortality in the United States. Results of this study demonstrate a significant positive correlation between PM concentration levels and COVID-19 mortality; however, this does not necessarily imply a causal relationship. These results are consistent with similar studies in Italy and China, where significant COVID-19 cases and corresponding deaths were exhibited. Furthermore, maps of the data demonstrate clustering of COVID-19 mortality which suggest further investigation into the social determinants of health impacting the pandemic.

## 1. Introduction

SARS-CoV-2 is a novel virus that was first reported in Wuhan, China in December of 2019. The virus causes the disease commonly known as COVID-19, which, since its emergence, has infected over 252 million individuals globally and taken the lives of more than 5 million in the same time span. As of 12 November 2021, the United States has experienced the highest number of cases, 46,868,744, with India just behind at 34,414,186, with cases still on the rise. Primary research on SARS-CoV-2 and COVID-19 was initially focused on understanding the biomolecular composition of the virus. This research has been used to develop multiple vaccines with great efficacy and antiviral treatments for the disease. The development of biomedical interventions has been crucial to combating this pandemic; now, we need to address the environmental confounding variables that could have exacerbated the pandemic. In this research study, we conducted a spatial analysis of particulate matter (PM) concentration and its association with COVID-19 mortality in the United States. Studies in Italy and China have explored this research question, in locations where high COVID-19 cases and corresponding deaths were exhibited. Few ecological studies concerning COVID-19 and PM had been conducted in the United States at the time of analysis (summer 2021), and this research will contribute to filling that void of knowledge and information.

Air pollution exists everywhere globally, although to a different extent, and concentrations of pollutants have been on the rise as the combustion of fossil fuels and burning of natural gas has increased. Natural events such as wildfires and volcanic eruptions can also contribute to air pollution levels. Consequently, health conditions, such as respiratory disease (RD), cardiovascular disease (CVD), nonfatal heart attacks, aggravated asthma, decreased lung function, increased infant mortality, and COPD, associated with ambient air pollution, cause 4.2 million deaths a year globally [1]. One of the primary pollutants associated with these illnesses is fine particulate matter.

Particulate matter is a combination of solid particles and liquid droplets that exist suspended in the air. These particles enter the atmosphere from either primary or secondary sources. Primary sources of particulate matter are particulates emitted directly from the source, and include construction sites, smokestacks, wildfires, and agricultural operations. Secondary sources of particulate matter are sources that emit precursors for particulate matter that undergo chemical/mechanical reactions in the atmosphere. Secondary sources of particulate matter include pollutants from vehicle emissions and power plants that include sulfur oxide, nitrous oxide, volatile organic compounds (VOCs), and ammonia [2]. Regulations on vehicle emissions and industrial pollutants to regulate the release of particulate matter precursors into the atmosphere has been beneficial in decreasing the concentration of particulate matter in the ambient air; however, concentrations are still above health regulations in some areas. In the United States (US), the Environmental Protection Agency (EPA) is the primary federal regulatory agency for protecting the environment and its interaction with human health. By order of the Clean Air Act, the EPA sets and enforces the National Ambient Air Quality Standards (NAAQS) for six common pollutants that have all been linked to negative human health outcomes when found in excess [3]. Evidence of the detrimental human health effects from particulate matter, one of the six common pollutants defined by the EPA, can be observed in historical case studies.

Between 1 December and 5 December 1930, in the Meuse River Valley, Belgium, individuals began reporting symptoms of larynx irritation, coughing, chest pain, and even foaming at the mouth. Within 24 h of the first reported symptoms, more than 60 individuals had died and many more were afflicted. Air pollution from numerous factories, in combination with a temperature inversion, trapped the particulate matter, creating a smog that killed many [4]. A brief 18 years later, in late October of 1948, the citizens of Donora and Webster, Pennsylvania experienced a nearly identical event. Citizens of the industrial region began mass-reporting respiratory distress, and by the end of the phenomenon, 20 citizens had died, 1440 were affected by serious illness, and another 1470 had mild to moderate symptoms. Subsequent studies of the Donora region have defined many long-term effects from the smog. A significantly excessive amount of CVD and cancer cases were reported in the decade following the smog event. In addition, core samples drawn from a nearby lake determined that after the opening of the Zinc Works industrial factory in 1915, a substantial increase in cadmium, lead, and zinc concentrations were found. Even after operations ceased, there was no visible reduction in the concentrations of cadmium or lead 70 years after the smog [5]. These contaminants are at high risk of being released into the water source if human disturbance or severe flood were to occur, subsequently causing another cascade of human health issues.

The health disasters that occurred in Donora and Webster initiated a chain of events that led to the passage of the Clean Air Act in the US; however, incidents continued to occur across global space. In December of 1952, London experienced one of the worst smog events ever recorded. It is estimated that there were approximately 12,000 excess deaths during the 5-day event and the subsequent 3-month period that can be directly tied to the particulate matter pollution. Similar to the previously discussed events, a temperature inversion occurred which allowed for minimal horizontal or vertical air movement near the ground. The colder temperature observed during this period exacerbated the pollution because domestic heat sources and power plants were functioning at maximum capacity to keep the citizens warm. In addition, London had just abandoned their electric trams for diesel-powered buses in July of 1952, which released an abundance of diesel emissions [6]. Traffic-related air pollutants (TRAPs) are still a large concern in the present day. A study conducted in Finland suggests that TRAPs severely impact the functioning and interaction of astrocytes (cells that regulate homeostasis, maintain functionality, and defend the central nervous system) and microglial cells (resident immune cells of the central nervous system). Results of the study suggest that TRAPs directly induce inflammation and neurodegeneration, which can lead to many other neurological diseases [7]. Case studies such as Meuse River Valley (Belgium), Donora (Pennsylvania), and London (England) demonstrate the importance of studying the relationship between particulate matter and human health and using the research to mitigate any future health and environmental disasters.

Previous research on particulate matter has demonstrated positive associations between particulate matter and mortality, especially mortality due to RD and CVD. A study conducted in 2003 explored the geographical variation in the effects of particulate matter on mortality. Using Bayesian statistics, the researchers divided the US into seven geographical regions (Northwest, Upper Midwest, Industrial Midwest, Northeast, Southern California, Southwest, and Southeast) and analyzed the concentrations of coarse particulate matter ≤ 10 μm (PM_10_) and daily cause-specific mortality data for the 90 largest cities (excluding Honolulu, HI and Anchorage, AK). The analysis concluded that daily variation of PM_10_ is positively associated with daily variation of mortality [8]. Although there was a modest variation in relative risk between the geographical regions, the stratification of the US in this manner does not consider geographical features, industries, and lifestyles that could impact the ecological environment, which impacts the onset and virulence of disease. Analyzing the US as a whole and then drawing regional differences can more effectively demonstrate and infer which geographical features, industries, and lifestyles are responsible for the increase in particulate matter and increased disease. In 2016, a similar research study analyzing the risk of asthma, myocardial infarction, and heart failure in association with fine particulate matter (PM_2.5_) concentration was conducted (defined as having an aerodynamic diameter ≤ 2.5 μm). Using advanced spatial epidemiological technologies, the research group used satellites and hierarchical Bayesian modeling to estimate PM_2.5_ concentrations in regions where ground monitors did not collect data. This methodology was used to study the geographical region of New York City, NY metropolitan and surrounding areas. This data collection method allowed the researchers to pull PM_2.5_ concentration data from multiple sources and overlay heat maps to obtain the most accurate and comprehensive PM_2.5_ data for the region. The use of heat maps provides great data visualization, and similar techniques are used in our research study to demonstrate where the association between COVID-19 and PM_2.5_ is high and low, “hot” and “cold”. In the study, the pollution data was tracked daily in concurrence with hospital admittance data for asthma, myocardial infarction, and heart failure, and the study concluded that high PM_2.5_ exposure is associated with increased risk of asthma, myocardial infarction, and heart failure [9].

Comparable conclusions were made about the association between human health risk and particulate matter in studies conducted in 2017 and 2018 in Beijing, China, and Verona, Italy, respectively. However, these studies looked specifically at long-term and continuous exposure to particulate matter. Both studies analyzed mortality specifically associated with CVD and RD. The Beijing study used a generalized additive model (GAM) to estimate excess risk for CVD and RD in relation to daily mean PM_2.5_ concentrations. The study stratified the results into four thresholds (75 μg/m³, 85 μg/m³, 105 μg/m³, and 115 μg/m³). The analysis concluded that individuals that were single, illiterate, and worked outdoors were more at risk for particulate matter-related CVD and RD than other demographic groups. Once the PM_2.5_ concentration reached 105 μg/m³ for nine consecutive days, the excess risk of CVD death for single, illiterate, or outdoor working individuals increased by as much as 45% [10]. This study supports the hypothesis that heavy industrial and mining regions may exhibit higher rates of COVID-19 mortality. In addition to infectious disease, individuals become more susceptible to chronic diseases, such as chronic obstructive pulmonary disease (COPD), in these regions, and chronic diseases can be comorbidities with COVID-19. In comparison, the study in Verona discovered that 11.3% of the total annual deaths due to CVD and RD from 2009 to 2014 can be attributed to PM_2.5_ pollution. The highest causes of mortality attributed to PM_2.5_ pollution were for ischemic heart disease and cerebrovascular disease (stroke). This study in Verona was unique because of its geographical location in the center of Italy’s Po Valley. The Po Valley is a highly industrial and agricultural region, and, in conjunction with the valley feature, is one of the most polluted regions in the world. Over the past decades, PM_2.5_ and PM_10_ concentrations there have decreased; however, concentration levels are still consistently above the European standard of 25 μg/m³, making particulate matter pollution an ongoing threat to human health in that region [11]. However, not all pollution is caused by anthropogenic activities. Pollution from natural events, such as wildfire, can also have adverse health effects, both chronic and associated with infectious disease.

In 2020, a study took place in Montana, USA to research the delayed effect of wildfire season particulate matter pollution on the subsequent influenza season. The study analyzed both short-lag (1–4 weeks) and long-lag (prior wildfire season months) effects on the following winter influenza season in Montana, USA. Using spatial regression models and a quasi-Poisson model, the study discovered no short-lag PM_2.5_ effect nor short-lag temperature effect on influenza. However, analysis did show that higher daily average PM_2.5_ concentrations during the wildfire season were positively associated with increased influenza in the following winter influenza season. Two different analyses of differing *p*-values (*p* = 0.04 and *p* = 0.008) demonstrated respective 16% and 22% increases in influenza rate per 1 μg/m³ increase in average daily summer PM_2.5_ [12]. This analysis of the impact of particulate matter on a viral respiratory disease in the US supports the hypothesis that forms our research question.

In addition to chronic and noninfectious diseases, particulate matter has been historically associated with infectious diseases, especially coronaviruses. Coronaviruses are a family of viruses that range from the common cold to severe acute respiratory syndrome (SARS). SARS, Middle Eastern respiratory syndrome (MERS), and COVID-19 are all in this family of coronaviruses. SARS first surfaced in 2002 and 2003 in Beijing, China. In comparison to COVID-19, SARS exhibited an extremely high global case fatality rate of up to 15%. Although more fatal, SARS could only spread from asymptomatic individuals, whereas individuals with COVID-19 can infect others several days prior to exhibiting any symptoms. This is one of the reasons why SARS-CoV-2, a very similar virus to SARS-CoV, has evolved from an epidemic to a pandemic. However, the biomolecular and symptomatic similarities of COVID-19 and SARS sparked similar ecological investigations into the relationship between ambient air quality and the virus.

A 2003 ecological study following the SARS epidemic found a strong correlation (correlation coefficient of 0.8568; *p*-value = 0.0636) between PM_10_ air pollution and SARS fatality. This study gathered air pollution index (API) and SARS fatality data from five geographical regions in China (Guangdong, Shanxi, Hebei, Beijing, and Tianjin). Relative risk (RR) and 95% confidence intervals were calculated for the data using Statistical Analysis Software (SAS). Final data analysis demonstrated APIs in Guangdong, Shanxi, Hebei, Beijing, and Tianjin during April and May to be 75, 95, 98, 99, and 104, and the corresponding case-fatality rates were 3.84%, 5.36%, 5.58%, 7.66%, and 8%, respectively. In addition, short-term and long-term PM exposures were analyzed, and both concluded an association between PM exposure and SARS fatality [13]. In 2005, a study focused on the region of Beijing, specifically, came to similar conclusions. In addition to analyzing the relationship between pollution and SARS fatality, this more recent study included meteorological variables (daily mean temperature, relative humidity, and dew point) collected from the State Meteorological Administration. Using a generalized additive model (GAM) with log link and Poisson error, the study produced relative risks that were statistically significant for PM_10_ and NO_2_ with a 4- or 5-day lag. However, the study did not find any significant association between daily SO_2_ concentration and SARS mortality with lags up to 6 days [14]. 

In the first year after COVID-19 emerged, ecological studies analyzing the relationship between particulate matter and COVID-19 mortality grew exponentially, especially in China and Italy. An Italian study looked at the spatial variation and patterns between PM_10_ and PM_2.5_ in comparison to COVID-19 incidence proportions and death rates using adjusted regression models. The study found that both fine and coarse particulate matter are positively associated with COVID-19 cases and deaths, and consequently explain the heterogenous distribution of COVID-19 cases and deaths in Italian provinces [15]. A similar study conducted in Wuhan, China used multivariate linear regression to conduct a temporal comparison between PM_10_ and PM_2.5_ concentrations with the case fatality rate (CFR) of COVID-19 in Wuhan. The temporal variation curves for particulate matter concentrations and COVID-19 CFR exhibited strong similarities. Results of the study found a positive relationship (*r* > 0.65, *p* ≤ 0.00003) between PM_10_ and PM_2.5_ concentrations and the COVID-19 CFR [16]. An additional meta-analysis offered an indirect impact of particulate matter on mortality of COVID-19 due to overexpression of ACE-2 receptors in the lungs. As explained in the article, the inhalation of large quantities of particulate matter causes inflammation in the lungs. This immune response stimulates several immune pathways, including the overexpression of ACE-2 receptors [17]. 

The research results found in Italy’s Po Valley and China correlating increased PM pollution to increased COVID-19 mortality prompted the study here. However, both of these prior studies focused on their respective geographic locations. Analysis of the PM relationship with COVID-19 mortality in the US was still relatively absent from literature at the conception of this study. Therefore, we were motivated to explore the spatiotemporal association between PM_2.5_ and COVID-19 mortality within the continental US and Hawaii (due to a lack of data consistency at the time of the analysis, Alaska was not included in the study population). 

## 2. Materials and Methods

To evaluate the association between COVID-19 mortality and PM_2.5_ concentration in the United States, county-level data was collected. COVID-19 data was collected from USAFacts on 7 June 2021. USAFacts collects the raw COVID-19 county data exclusively from approximately 70 different government agencies, including varying commissions, bureaus, and departments. Raw COVID-19 case numbers and total deaths were provided for each individual county [18]. The mortality rate per 10,000 for each county was calculated in MS Excel (Microsoft Corporation, Redmond, WA, USA) using Equation (1).

(1) $$\left(\frac{\mathrm{Total}\;\mathrm{Deaths}}{\mathrm{Population}}\times 10,000\right)$$

PM_2.5_ data was collected from the CDC National Environmental Public Health Tracking Network for the year 2016, the most recent annual data [19]. Population data for each county was collected from the United States Department of Agriculture (USDA) Economic Research Service. The USDA county population data was derived from the United States Census Bureau and was last updated on 13 May 2020 [20]. Alaskan counties were excluded from this study due to a lack of particulate matter data at the time of the study.

All data was organized in Excel, and repopulated into SPSS (IBM Corporation, New York, NY, USA) for analysis. Descriptive statistics (N, minimum, maximum, mean, standard deviation) were calculated in SPSS for all variables. Next, a Kolmogorov–Smirnov (K–S) test was conducted to check for normality in the dataset using Equation (2).

(2) $$D=Maximum\left|F_{o}\left(X\right)-F_{r}\left(X\right)\right|$$

To analyze the correlation between the variables we then utilized both a Spearman’s rho and Kendall’s tau analysis. The equations for each, respectively, are represented below.

(3) $$\rho =1-\frac{6\sum d_{i}^{2}}{n\left(n^{2}-1\right)}$$

(4) $$t_{b}=\frac{P-Q}{\sqrt{\left(P+Q+X_{0}\right)\left(P+Q+Y_{0}\right)}}$$

A Spearman’s rho correlation test is for nonparametric ordinal, interval, or ratio data. This test determines the strength and direction of a correlation assuming a monotonic relationship between the two variables. Similarly, a Kendall’s tau rank correlation test was conducted due to the nonparametric nature of the dataset. Kendall’s tau assumes that the variables are either continuous or ordinal, and that the variables have a monotonic relationship. Following the primary data analysis, the Excel 3D Maps tool was used to create a data visualization. A gradient color scale was used to depict the varying levels of COVID-19 mortality and PM_2.5_ annual average concentration levels, similar to a heat map. The maps were stratified by county, and each map represents the spatial distribution of an individual variable. 

## 3. Results

All variable correlations were statistically significant at the 0.01 alpha level (2-tailed), as depicted in Table 1 and Table 2. Considering the large sample of 3110 counties included in this study, these results demonstrate a significant association between COVID-19 mortality and fine particulate matter concentrations. 

**Figure 1:**
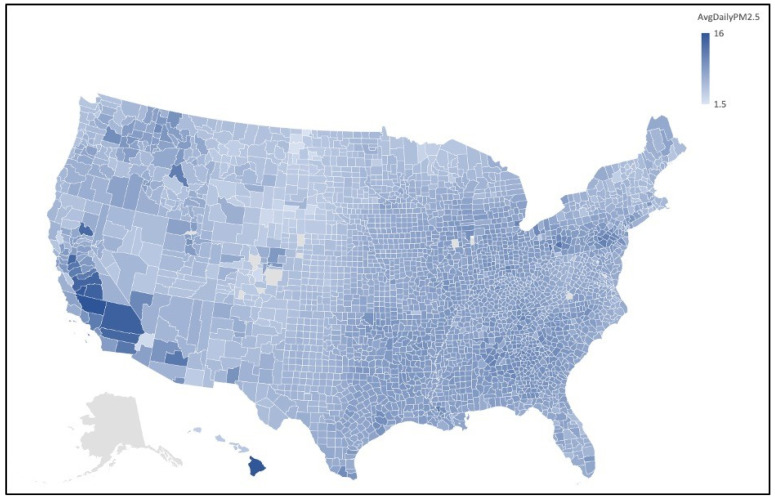
Average daily PM_2.5_ map of U.S. counties. Map of the average daily PM_2.5_ for each country in the United States, excluding Alaska. Clusters of higher PM_2.5_ concentration can be seen in the Detroit, MI and Philadelphia, PA regions, California’s Central Valley, and in Hawaii.

**Figure 2:**
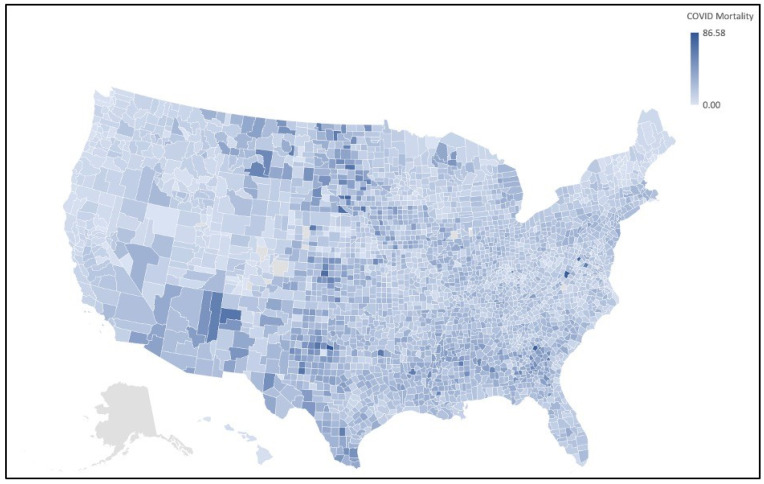
COVID-19 mortality totals of U.S. counties map. Map of the COVID-19 mortality rate per 10,000 by county in the United States, excluding Alaska. Clusters of high COVID-19 mortality can be observed in the Upper Midwest, Southwest, and South.

Some correlation coefficients of particular interest include Kendall’s tau correlation analysis between COVID-19 deaths and daily PM_2.5_ annual average (*r* = 0.309), Spearman rho correlation analysis between COVID-19 deaths and daily PM_2.5_ annual average (*r* = 0.440), and Spearman rho correlation analysis between COVID-19 cases and daily PM_2.5_ annual average (*r* = 0.365). From the results of the statistical analysis and visual interpretation of the maps, we can conclude that there is a significant positive correlation between COVID-19 mortality rate and PM_2.5_ concentration. Figure 1 and Figure 2 depict the regions of high and low COVID-19 mortality rates and PM_2.5_ concentration for all counties in the United States, excluding Alaska. 

In addition to the general study that encompassed all United States counties (minus Alaska), we conducted a substudy to identify clusters of high COVID-19 mortality and high PM_2.5_ concentration. To complete this study, the particulate matter data was stratified into low, medium, and high groups. At least 30 data points were selected from each strata and depicted via Excel 3D maps. 

**Figure 3:**
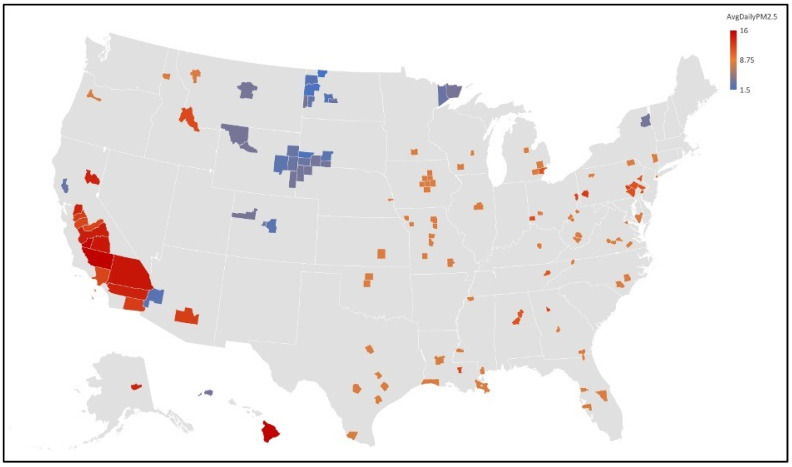
Substudy average daily PM_2.5_ map of U.S. counties. Map of particulate matter concentration levels by county for the stratified substudy. High PM_2.5_ concentrations are concentrated in California’s Central Valley and Hawaii. The middle strata are evenly dispersed across the eastern United States, and low PM_2.5_ levels are concentrated in the Upper Midwest.

**Figure 4:**
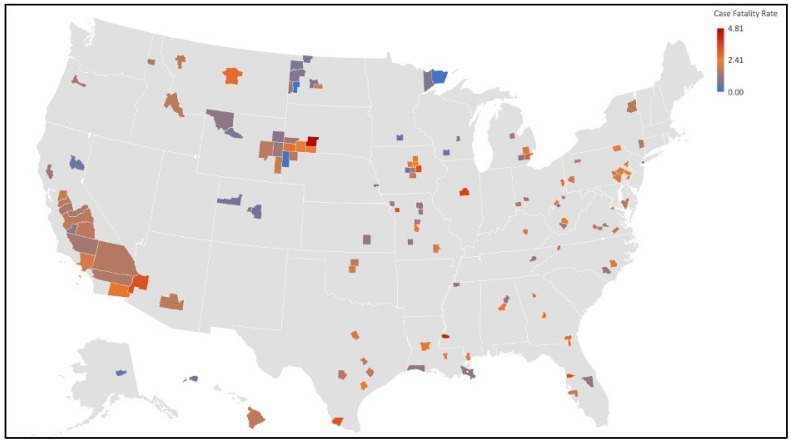
Substudy case fatality rate of U.S. counties map. Map of case fatality rates by county for the stratified substudy. High CFRs are observed in California’s Central Valley in addition to the Upper Midwest where low PM_2.5_ concentrations were observed.

Results of a Pearson correlation found that in the high strata, there was a significant negative correlation between the CFR and PM_2.5_ as well as the mortality rate and PM_2.5_ at the 0.05 level. This analysis contradicts the findings of the overall study. Analyzing the maps in Figure 3 and Figure 4, we can see that counties in the Upper Midwest, where PM_2.5_ is low, inversely have high case fatality rates from COVID-19. The rural nature of the region opens opportunities for further investigation into the level of access to adequate healthcare and other social determinants of health that could influence this inverse relationship between PM_2.5_ concentrations and COVID-19 CFR in the region.

## 4. Discussion

Analysis of the maps created in our study demonstrated small clusters of counties for both COVID-19 mortality and PM_2.5_ concentrations. The first group of clusters that indicates an association between air pollution and COVID-19 mortality is located in the Upper Midwest (Iowa, Nebraska, Wyoming, South Dakota, and North Dakota). The small clusters in this region suggest possible associations with social determinants of health in terms of mortality correlations. The rural environment that exists in this region contributes negatively to many of the social determinants of health. Poverty, proximity to healthcare facilities, and health literacy are all associated with rural environments and are all additional variables that could contribute to increased mortality rate in their respective counties. In addition, the Upper Midwest is home to multiple Native American reservations. The Native American population is more vulnerable to COVID-19 due to the “prevalence of underlying chronic health conditions, lack of institutional resilience, the relationship between the federal government and tribal governments, and lack of social trust” [21]. Additionally, a CDC Morbidity and Mortality Weekly Report (MMWR) suggested that American Indian and Alaska Native (AI/AN) persons experience a COVID-19 incidence 3.5 times higher than their white neighbors. However, this elevated incidence is influenced by other socioeconomic factors that could possibly facilitate the spread of the virus, such as reliance on shared transportation, lack of running water, and large household size (multigenerational households) [22]. Similar cluster patterns can be seen in the Southwest, where Native American reservations are also prevalent. 

Clusters of counties were also demonstrated on the map depicting elevated PM_2.5_ concentrations. Two areas with correlated clusters were in the Detroit, MI, and Philadelphia, PA cities and surrounding counties. Detroit is an urban center that has a large population. In addition to traditional urban density of activity, Detroit is a global hub for many industries. Some of the highly polluting industries in Detroit include: an oil refinery, a steel mill, a wastewater treatment plant, and coal- and gas-fired power plants [23]. Similarly, Philadelphia, the location of a cluster on our map, is an industry-packed city. 

One final, and greatly significant, spatial anomaly demonstrated on the PM_2.5_ map is the extremely high concentration levels in the counties of California’s Central Valley. The valley’s geographical feature has been associated with climate inversion events that trap air in the valley, leading to increased pollution and stagnant air [24]. Similar climatology is exhibited in Italy’s Po Valley, one of the hot spots for COVID-19 early in the pandemic [25]. Research conducted by Zlatev and his associates has demonstrated that as climate change progresses and temperatures rise, corresponding air pollution levels will rise concurrently [26,27]. If this trend continues, living in valley regions such as Central Valley and Po Valley will be a severe comorbid exposure for any kind of respiratory illness.

Prior to the study, we did not anticipate that Hawai’i County would have some of the highest levels of annual particulate matter pollution. After further investigation, we discovered that, whereas Honolulu and Maui have some of the cleanest air in the country, volcanic activity from Kīlauea emits enormous amounts of PM_2.5_ and sulfur dioxide into the air [28]. Interestingly, even with such high levels of PM_2.5_, the COVID-19 mortality rates in Hawai’i were well below the national average and what would be expected based on the results of this study. It is unclear why Hawai’i County is an outlier in this study, but confounding variables, such as population density, lifestyle habits, isolation of the island, and medical practices, could all impact the COVID-19 mortality rate. Some recent studies have found associations between vitamin D levels and COVID-19. Current research suggests that those with higher levels of vitamin D, which is to be expected in the Hawaiian population, have generally better COVID-19 outcomes. In fact, the Northern Hemisphere has borne most of the burden of cases and deaths for COVID-19, with the exception of the Nordic countries [29,30]. Furthermore, it is evident that individual factors, such as exercise, diet, and vitamin D exposure, in addition to structural policy issues, such as essential worker exposure, blue- vs. white-collar exposure, and nonuniversal healthcare coverage, concurrently influence an individual’s risk of contracting, and possibly dying from, the COVID-19 virus [31,32,33,34,35]. Moreover, individual risk factors related to age [36] and obesity [37] have also been associated with severity of COVID-19 disease.

Based on the methodology of this research, we cannot attribute causation to these variables; however, the results of this study and similar studies in the US, China, and Italy warrant further investigation into a possible association between high PM_2.5_ concentrations and increased COVID-19 mortality rates.

## 5. Conclusions

The results of this study add valuable information to the ever-growing data on COVID-19. However, the study does raise many questions and variables for further investigation. The clustering of high mortality rates in rural regions in the Upper Midwest and somewhat in the Southwest and South allude to the intervention of social determinants that could influence health outcomes. Specifically, social and healthcare structures on Native American reservations that are present in the Upper Midwest and Southwest regions may have been associated with the anomalously high COVID-19 mortality observed in rural counties colocated there. Investigations on how to improve health systems and communication on these reservations would be suggested in order to prepare for any future epidemics. Additionally, the high PM_2.5_ concentrations and low mortality rates on the island of Hawaii warrant further investigation. Individual risk factors related to age, obesity, diet, vitamin D levels, and physical activity are all variables that could influence the inverse relationship between COVID-19 mortality and PM_2.5_ concentrations on the island.

## Figures and Tables

**Table 1:** Kendall’s tau correlation analysis.

	COVID-19 Cases	COVID-19 Deaths	COVID-19 Mortality Rate	Daily PM_2.5_ Annual Average
COVID-19 Cases	1.000	0.785	−0.064	0.254
COVID-19 Deaths	0.785	1.000	0.124	0.309
COVID-19 Mortality Rate	−0.064	0.124	1.000	0.140
Daily PM_2.5_ Annual Average	0.254	0.309	0.140	1.000

All correlations are statistically significant at the 0.01 alpha level.

**Table 2:** Spearman’s rho correlation analysis.

	COVID-19 Cases	COVID-19 Deaths	COVID-19 Mortality Rate	Daily PM_2.5_ Annual Average
COVID-19 Cases	1.000	0.931	−0.085	0.365
COVID-19 Deaths	0.931	1.000	0.190	0.440
COVID-19 Mortality Rate	−0.085	0.190	1.000	0.203
Daily PM_2.5_ Annual Average	0.365	0.440	0.203	1.000

All correlations are statistically significant at the 0.01 alpha level.

## Data Availability

Publicly available datasets were analyzed in this study. This data can be found here: https://usafacts.org/visualizations/coronavirus-covid-19-spread-map/ (accessed on 7 June 2021); https://data.ers.usda.gov/reports.aspx?ID=17827 (accessed on 9 June 2021); https://ephtracking.cdc.gov (accessed on 1 June 2021).
